# Additional value of biomechanical indices based on CTa for rupture risk assessment of abdominal aortic aneurysms

**DOI:** 10.1371/journal.pone.0202672

**Published:** 2018-08-22

**Authors:** Eva L. Leemans, Tineke P. Willems, Cornelis H. Slump, Maarten J. van der Laan, Clark J. Zeebregts

**Affiliations:** 1 Departments of Surgery (Division of Vascular Surgery), University Medical Center Groningen, University of Groningen, Groningen, The Netherlands; 2 Departments of Biomechanical Engineering and Physics, Amsterdam UMC, University of Amsterdam, Amsterdam, The Netherlands; 3 Radiology, Amsterdam UMC, University of Amsterdam, Amsterdam, The Netherlands; 4 Department of Robotics and Mechatronics, MIRA Institute for Biomedical Engineering and Technical Medicine, University of Twente, Enschede, The Netherlands; 5 Radiology, University Medical Center Groningen, University of Groningen, Groningen, The Netherlands; Hospital Universitari Bellvitge, SPAIN

## Abstract

**Objective:**

Biomechanics for rupture risk prediction in abdominal aortic aneurysms (AAA) are gaining popularity. However, their clinical applicability is still doubtful as there is lack of standardization. This study evaluates the added value of biomechanical indices in rupture risk assessment.

**Methods:**

This study included 175 asymptomatic, 11 sAAA and 45 ruptured aneurysms. 3D-geometries were reconstructed using computer tomography angiographies. Subsequently, finite element models were made to calculate peak wall stress (PWS), peak wall rupture index (PWRI) and the rupture risk equivalent diameter (RRED). The indices were determined with a dedicated software to facilitate standardization.

**Results:**

SAAAs showed a trend towards higher PWS, PWRI and RRED compared to asymptomatic AAAs, but PWS (22.0±5.8 vs. 33.4±15.8 N/cm^2^), PWRI (0.52±0.2 vs. 1.01±0.64), and RRED (65±60 vs. 98±51 mm) were significantly (p = 0.001) higher in ruptured. However, after diameter-matching no significant differences were seen. The ROC-curves for the maximum diameter and all biomechanical indices were similar but it slightly increased when diameter and biomechanical indices were combined.

**Conclusions:**

This study showed no added value for biomechanical indices in AAA rupture risk assessment. Additionally, the difficulty of such an assessment increases. However, as symptomatic aneurysms show a trend towards higher biomechanical indices with similar diameters the indices may provide information about aneurysm growth and development.

## Introduction

Abdominal aortic aneurysms (AAAs) occur in approximately five percent of the population and are c potentially life threatening in case of rupture.[1] Unruptured AAAs could be repaired with open or endovascular surgery in an elective setting, but both carry a risk for complications. The decision to intervene is usually based on the clinical condition of the patient, medical imaging, and patient preference. Currently, the maximum aneurysm diameter and expansion rate are the two most important risk factors for AAA rupture. Both have been extensively validated.[2,3] In general, small (maximum diameter below 5.5 cm) and slowly expanding (<0.3cm per year) aneurysms are less likely to rupture. However, aneurysm diameter is a population based risk estimate and thus some small aneurysms rupture while some large aneurysms remain stable.[3–5] Furthermore, small aneurysms are more prevalent than large aneurysms.[6,7] Although the risk is lower in small aneurysms the small fraction of this majority may be a significant part of the ruptured AAAs. Thus there is a clear need for a patient tailored approach and patient-specific decision making.

Diagnostic indices which are able to accurately estimate the rupture risk of a specific aneurysm would greatly improve this patient-specific assessment. For this purpose biomechanical indices were developed.[8–11] These indices are based on the basic principle of material failure; an aneurysm ruptures when wall stress exceeds wall strength. Therefore, they might relate more closely to the pathological process of growth and rupture than the maximum diameter. These indices are extracted using computational models and diagnostic imaging such as ultrasound, computed tomography scanning or magnetic resonance imaging.[12,13] Hereby, several patient-specific biomechanical indices are calculated, such as peak wall stress (PWS), peak wall rupture index (PWRI) and rupture risk equivalent diameter (RRED). PWS is the maximum in plane wall stress. The wall strength is estimated using patient characteristics, subsequently, PWRI can be calculated by dividing calculated wall stress by the estimated wall strength.[10,14,15] The RRED reflects the PWRI translated to equivalent diameters of the average aneurysm patient.[16]

The clinical applicability and additional value of these biomechanical indices compared to the maximum diameter are still unknown, particularly, due to the lack of standardization.[17] However, over recent years dedicated software for the biomechanical analysis of AAA became available. This software might help to implement the biomechanical analysis in daily clinical practice. Previous studies showed that it has low inter- and intra-observer variability.[18]

The aim of this study was to assess the clinical value of a biomechanical analysis method using a patient-specific AAA geometry and finite element analysis. The clinical usability is assessed by comparing the resulting geometric and biomechanical indices between asymptomatic, symptomatic non-ruptured and ruptured AAAs.

## Methods

### Study design

The study was approved by the Institutional Review Board (METc2016.161). The requirement for informed consent was waived because no diagnostics other than routine clinical imaging were used in this study.

Patients were retrospectively collected between January 2003 and December 2014 from a prospectively held database containing acute and elective endovascular treated patients at the vascular surgery division of a large tertiary referral center. Acute repair was performed in case of rupture or symptomatology.

### Patient selection and characteristics

**Fig 1:**
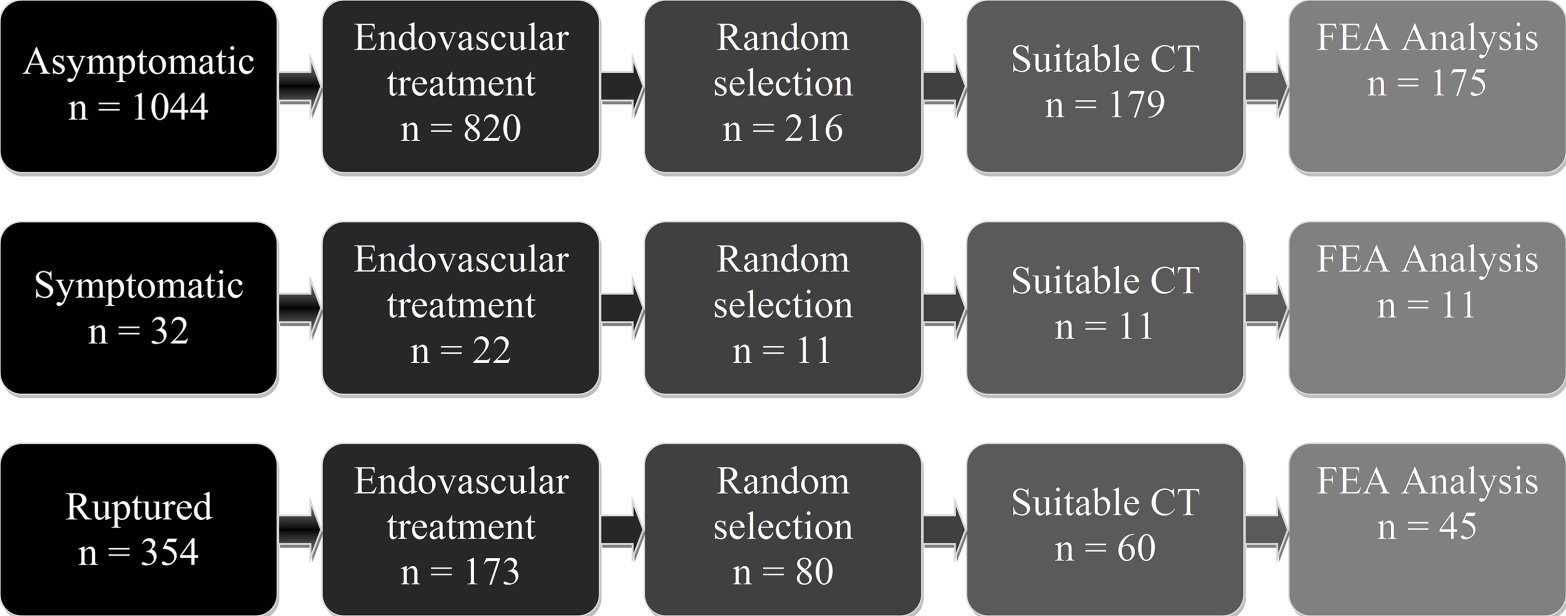
Flowchart patient selection.

Primary selection consisted of 216 asymptomatic, 11 symptomatic intact and 80 ruptured patients with a non-inflammatory infrarenal AAA which are randomly selected form the database using simple random sampling. An extension to the common iliac arteries was accepted (EUROSTAR classification type D)[19]. Only patients with a suitable preoperative computerized tomography angiography (CTa) were included. The CTa was deemed suitable when the aorta was visible from the renal arteries to the iliac bifurcation, and when the lumen was distinguishable from the intraluminal thrombus. Fig 1 displays the flow diagram for the selection of patients. Older CTa often have thicker slices and less contrast between lumen, thrombus and surroundings. Therefore, only a small part of the scans before 2010 were deemed suitable (5% of the CTa after 2010 were excluded versus 12.5% of the CTa before 2010).

The symptomatic non-ruptured group (sAAA) contained patients who presented with AAA associated symptomatology, such as abdominal and/or back pain, but had no signs of rupture on CTa. These patients underwent endovascular repair after ruling out other differential diagnoses. The ruptured group (rAAA) contained patients who presented with acute signs of rupture which were confirmed on preoperative CTa. Notably, the post-rupture scans were used for the ruptured group.

The following risk factors and co-morbidities were registered with definitions according to the guidelines of the American Heart Association[20]: age, sex, diabetes mellitus (yes/no), smoking habits (current smoker yes/no), blood pressure (systolic, diastolic and mean arterial pressure (MAP; $\frac{1}{3}$ systolic pressure + $\frac{2}{3}$ diastolic pressure), body mass index (BMI) and an early family history for AAA (male <55 years of age, female <65 years of age (first-degree relative)), hypercholesterolemia (diagnosis yes/no, (preventative) statins yes/no), and other cardiovascular disease (CVD; stroke, coronary/peripheral artery disease). Clinical data were collected from the last measurement in a non-critical setting within one year before intervention, either during routine check-ups or at hospital admission.

### Biomechanical analysis

**Fig 2:**
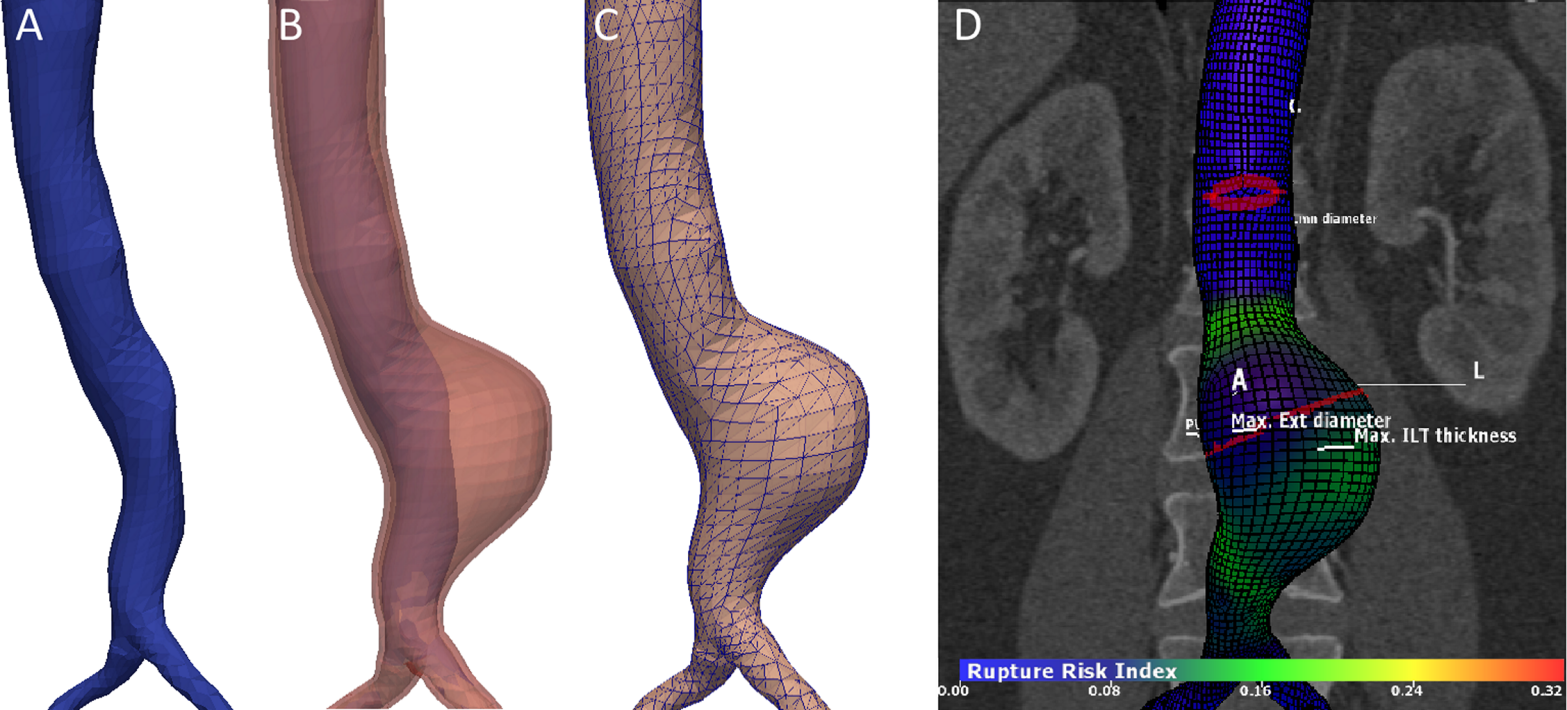
Workflow of the used software. A. Luminal segmentation in blue B. Luminal (blue) and thrombus (red) segmentation C. Exterior mesh. D. PWRI outcome displayed on the 3D model.

All biomechanical analyses were performed using commercially available software (A4research™, VASCOPS, Graz, Austria). The software uses several steps. First, AAA geometry is reconstructed by segmenting the vessel lumen, the intraluminal thrombus (ILT), and the vessel wall (Fig 2).[12] Second, a mesh is generated and the finite element analysis (FEA) is executed. Details are described below.

For an accurate segmentation several requirements have to be met. In 4 AAA and 15 RAAA biomechanical analysis was not possible due to limited amount of contrast (n = 4), insurmountable distinction between ILT and hematoma (n = 3) and complex geometries resulting in intersecting faces (n = 12). Consequently, these cases were excluded. A total of 179 asymptomatic AAAs (aAAA), 11 symptomatic AAAs (sAAA) and 60 ruptured AAAs (rAAA) cases were included.

#### Geometry reconstruction

Segmentation was semi-automatic using deformable snake and balloon models for the 2D and 3D segmentation, respectively. These are objects that deform within the image until they stop at the boundary of a structure (lumen or vessel). The evolution of the object depended on a set of reconstruction parameters and contrast differences. First, a snake model to pre-segment the luminal surface was initialized by manually placing an initialization circle in the lumen of the iliac arteries. Subsequently, the luminal service was perfected using a balloon model and the exterior surface was segmented with a second balloon starting from the luminal surface. Segmented volumes were manually corrected through enriching image data and control polygons. The amount of user interaction depended on the image quality and the complexity of the aneurysm. In general ruptured aneurysms required more manual correction. Finally, the external vessel wall and ILT were automatically segmented by the software.

#### Mesh generation and finite element analysis

After geometry reconstruction a mesh of the 3D volume containing hexahedral elements was created automatically by the software. Details are presented elsewhere.[12] The FEA region was set from the renal arteries to just distal to the iliac bifurcation. The model was pressurized at the MAP. In case the blood pressure (BP) was not reported a set BP of 140 over 80 mmHg was used (n = 8, 2, 12 for aAAA, sAAA and rAAA cases, respectively). An isotropic constitutive model was used for the ILT and aneurysm wall.[21] The wall strength is estimated using the position of the ILT, sex, family history and the relation between the local diameter and the calculated normal aortic diameter.

#### Parameter calculation

Several geometric (maximum diameter, volume) and biomechanical parameters (PWS, PWRI, RRED) were extracted. PWS is produced by the blood pressure resulting in an in-plane wall stress and consequently deformation. The PWRI is a calculated index, dividing the calculated wall stress through the estimated wall strength. The latter (RRED) expresses the diameter of an average AAA that has the same PWRI. All parameters were calculated automatically. The maximum diameter was based on measurements perpendicular to the center luminal line. The software produced colored overlays to provide information of the distribution of wall stress and rupture risk (Fig 2). The maximum diameter as determined by an experienced radiologist, using also measurements perpendicular to the center luminal line, was extracted from the patient file to assess the differences with current clinical practice.

### Statistical analysis

Statistics were expressed as mean ± standard deviation in case of a normal distribution. Percentages were given for nominal variables. Normality of the data was tested using the one sample Kolmogorov-Smirnov test. Comparison between scans or groups was done using a Student t-test in case of normal distribution. Mann-Whitney U tests were performed to compare skewed variables. Dichotomous variables were compared using the chi square test. Missing values were pair wise excluded. To evaluate the capacity to correctly predict rupture on the basis of the diameter and biomechanical indices a ROC analysis was performed, examining the discrimination between groups under varying thresholds. A combined model of all biomechanical indices (PWRI, PWS and RRED) and the maximum AAA diameter was made using logistic regression. Subsequently, the predicted probability was included into the ROC-analysis. All statistical analyses were performed using SPSS statistics 23 (IBM, New York, NY, United States). Significance was set at p < 0.05. To minimize the false discovery rate we applied Bonferroni correction for multiple analyses on the same dependent variable. The adjusted significance levels was of .017 per test (.05/3).

#### Diameter matching

A sub analysis comparing only size-matched AAA and RAAA subjects was done. Hereby, providing a more stringent analysis of whether the biomechanical outcomes could differentiate between asymptomatic AAA and RAAAs. The diameters as measured by the software were matched using SPSS Case-Control matching. Match tolerances were set at 5 mm, resulting in 31 matches within the tolerance rate.[22]

## Results

All three groups had similar demographic characteristics. No significant differences were observed (Table 1, P > 0.017). The AAA group showed a skewed distribution of the maximum diameter as most values were between 50 and 60 mm due to the current threshold to treat.

**Table 1:** Demographic variables for aAAA, sAAA and rAAA.

*Variable*	*AAA*	*SAAA*	*RAAA*	*P-value*
N	175	11	45	-
Age (year)	72.4 ± 8.7	73.4 ± 10.4	73.9 ± 8.7	0.71^a^/0.40^b^/0.95^c^
Male	156 (89%)	7 (63%)	39 (87%)	0.01^a^/0.64^b^/0.07^c^
Blood pressure (mmHg) (n = 209)				
Systolic	135 ± 18	136 ± 13	140 ± 25	0.79^a^/0.29^b^/0.64^c^
Diastolic	76 ± 12	79 ± 5	76 ± 16	0.09^a^/0.23^b^/0.40^c^
MAP	96 ± 13	98 ± 6	96 ± 20	0.22^a^/0.86^b^/0.54^c^
BMI (n = 178)	26.9 ± 4.2	23.9 ± 3.1	26.6 ± 6.9	0.20^a^/0.89^b^/ 0.91^c^
Diabetes mellitus	23 (13%)	1 (9%)	7 (15%)	0.70^a^/0.67^b^/0.58^c^
Cardiovascular disease	107 (61%)	9 (82%)	24 (51%)	0.17^a^/0.14^b^/0.05^c^
Smoking (n = 211)	86 (49%)	3 (27%)	22 (47%)	0.16^a^/0.98^b^/0.20^c^
Hypercholesterolemia (n = 152)				0.40^a^/0.15^b^/0.92^c^
Diagnosis	31(18%)	3 (27%)	10 (21%)	
Preventative medication	66 (38%)	2 (18%)	11 (23%)	
Positive family history (n = 21)	10 (5%)	1 (9%)	0 (0%)	0.38^a^/0.14^b^/0.08^c^

BMI = body mass index. MAP = mean arterial pressure, AAA = asymptomatic abdominal aortic aneurysm, SAAA = symptomatic non-ruptured abdominal aortic aneurysm, RAAA = ruptured abdominal aortic aneurysm.

a: AAA compared to SAAA

b: AAA compared to RAAA

c: SAAA compared to RAAA

### Evaluation biomechanical indices and diameter

The sAAA group showed similar geometric values compared to the aAAA group (P > 0.0017). However, a small but insignificant trend towards higher biomechanical indices in the sAAA group was seen (P = 0.08, 0.16, 0.20 for PWS, PWRI and RRED, respectively).

All geometric and biomechanical indices were significantly higher in the rAAA group compared to the aAAA group (P < 0.001), for instance PWS was 22.0 ± 5.8 vs. 33.4 ± 15.8 N/cm^2^ for the aAAA and rAAA, respectively (Table 2).

**Table 2:** FEA outcomes of aAAA, sAAA and rAAA.

	*AAA*	*SAAA*	*RAAA*	*P-value*
n	175	11	45	-
Maximum diameter by radiologist (mm)	60 ± 11	56 ± 9	77 ± 19	0.37^a^/0.001^b^/ 0.001^c^
Maximum diameter by software (mm)	63 ± 13	64 ± 14	88 ± 24	0.88^a^/0.001^b^/0.002^c^
Total luminal volume (cm^3^)	93 ± 49	93 ± 56	190 ± 134	0.90^a^/0.001^b^/ 0.012^c^
Total volume (cm^3^)	200 ± 102	195 ± 64	424 ± 214	0.80^a^/0.001^b^/0.001^c^
Total ILT volume (cm^3^)	83 ± 61	65 ± 35	186 ± 135	0.49^a^/0.001^b^/0.001^c^
PWS (N/cm^2^)	22.0 ± 5.8	24.3 ± 5.4	33.4 ± 15.8	0.08^a^/0.001^b^/ 0.04^c^
PWRI	0.5 ± 0.2	0.6 ± 0.3	1.0 ± 0.6	0.16^a^/0.001^b^/ 0.06^c^
RRED (mm)	65 ± 60	67 ± 24	98 ± 51	0.20^a^/0.001^b^/0.07^c^

ILT = intraluminal thrombus, PWS = peak wall stress, PWRI = peak wall rupture index, RRED = rupture risk equivalent diameter, AAA = asymptomatic abdominal aortic aneurysm, SAAA = symptomatic non-ruptured abdominal aortic aneurysm, RAAA = ruptured abdominal aortic aneurysm.

a: AAA compared to SAAA

b: AAA compared to RAAA

c: SAAA compared to RAAA

Significant differences were also seen in geometric indices sAAA and the rAAA group (P<0.017). For instance the total ILT-volume was significantly higher in the rAAA; 65 ± 35 cm^3^ versus 186 ± 135 cm^3^ (P = 0.001) for the sAAA and rAAA, respectively. However the biomechanical indices did not show a significant difference after Bonferroni correction. For instance PWS was 24.3 ± 5.4 vs. 33.4 ± 15.8 N/cm^2^ (P = 0.04) for the sAAA and rAAA, respectively (Table 2).

An overlap was seen between the aAAA and SAAA cases for both the diameter and biomechanical indices. However, a larger overlap was seen between the aAAA and rAAA groups. Thus several ruptured cases could not be distinguished from the asymptomatic cases based on these parameters.

### Diameter matching

After diameter matching the demographic characteristics were still similar (P > 0.05) between the aAAA and rAAA group. However, no differences in biomechanical indices were seen between the aAAA and the rAAA group after diameter matching (Table 3; P > 0.05).

**Table 3:** FEA outcomes of the diameter matched subgroup.

	*AAA*	*RAAA*	*P-value*
n	31	31	-
Maximum diameter by radiologist (mm)	71 ± 15	72 ± 18	0.81
Maximum diameter by software (mm)	77 ± 16	78 ± 17	0.67
Total luminal volume (cm^3^)	132 ± 79	132 ± 76	0.76
Total volume (cm^3^)	296 ± 150	324 ± 148	0.34
Total ILT volume (cm^3^)	129 ± 91	158 ± 117	0.34
PWS (N/cm^3^)	26.1 ± 8.9	26.2 ± 7.5	0.99
PWRI	0.69 ± 0.33	0.70 ± 0.27	0.61
RRED (mm)	88 ± 89	73 ± 22	0.95

ILT = intraluminal thrombus, PWS = peak wall stress, PWRI = peak wall rupture index, RRED = rupture risk equivalent diameter, AAA = asymptomatic abdominal aortic aneurysm, SAAA = symptomatic non-ruptured abdominal aortic aneurysm, RAAA = ruptured abdominal aortic aneurysm.

### ROC analysis

**Fig 3:**
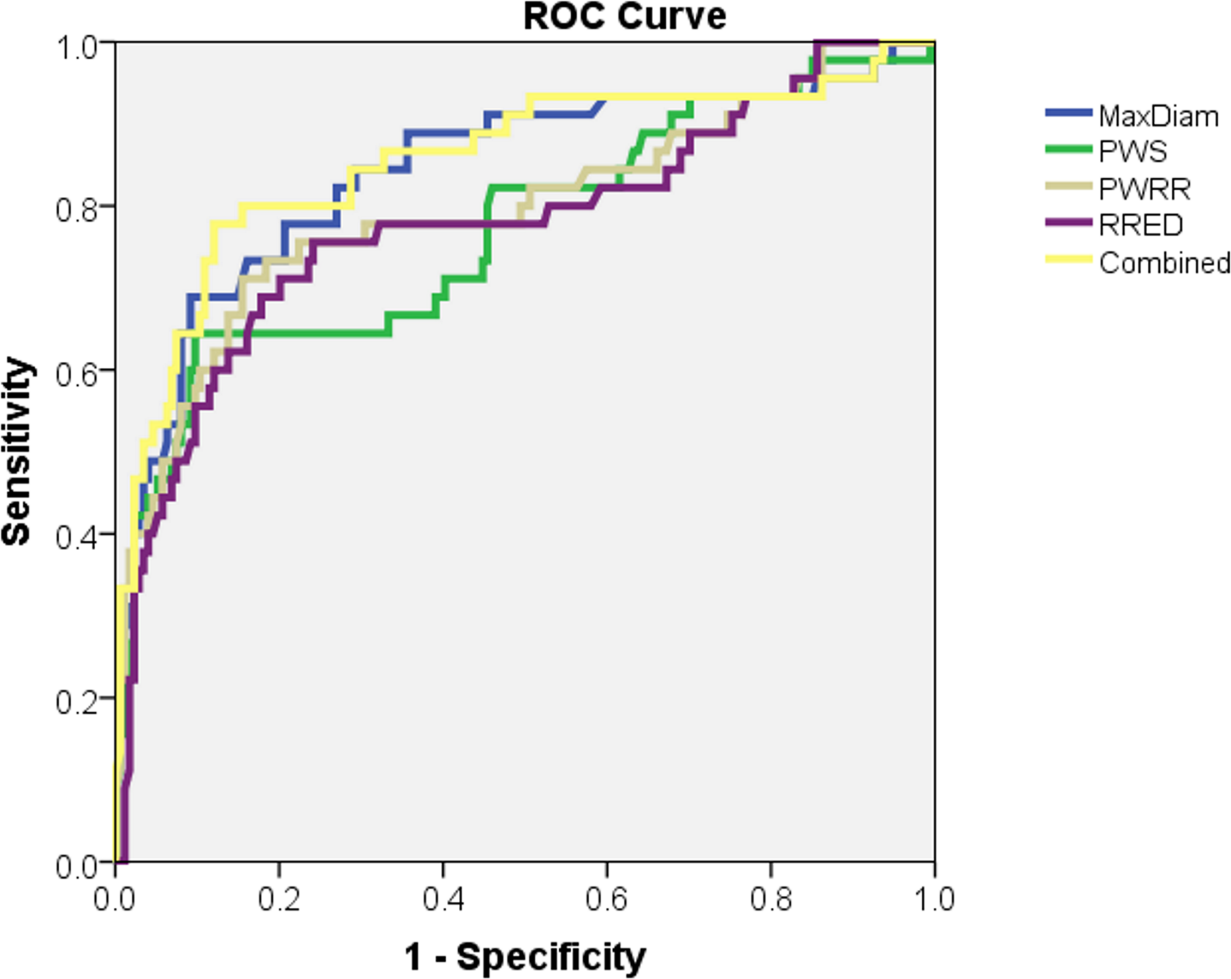
ROC-curve for the ability to accurately predict rupture. Maximum diameter (blue), PWS (green), PWRI (brown), RRED (purple) and combination of all parameters (yellow).

The ROC-curves are displayed in Fig 3. The maximum diameter shows a slightly higher area under the curve (AUC) compared to the biomechanical indices; 0.843, 0.770, 0.796, and 0.778 for the maximum diameter, PWS, PWRI and RRED, respectively. However, when the maximum diameter was combined with the biomechanical indices the AUC increased a little bit to 0.855.

## Discussion

This study examined three potential biomechanical parameters (PWS, PWRI and RRED) for rupture risk prediction of AAA based on FEA with patient specific geometries segmented from CTa. SAAAs showed a trend towards higher values of these biomechanical indices compared to aAAA while no significant difference in maximum diameter was seen. This trend suggests that biomechanical indices ameliorate rupture risk prediction compared to the maximum diameter alone as symptomatic aneurysms are prone to rupture.[23,24]

The results showed significant higher biomechanical indices in rAAA which is consistent with previous studies.[9,10,11,15,25–30,31] However, the diameter was also significantly higher. After diameter matching no significant differences in geometric or biomechanical parameters between aAAA and rAAA were seen. Three out of four previous studies using diameter matching did show a significant difference between these groups.[9,11,28,30] These studies excluded the large and small diameters to create a diameter matched group (55-75mm). Our study used a true matched subject design as ruptured aneurysms were matched to similar sized aneurysms in the aAAA group, giving a more accurate overview of the total AAA population.

Biomechanical indices are a direct result of several clinical factors such as age, sex, blood pressure, morphology and shape. Therefore, these indices might facilitate the rupture risk estimation of a specific patient. Additionally, the number of patients with small AAAs expands with the introduction of screening programs, increasing the need to identify the small AAAs at risk of rupture.[32] In this group biomechanical indices might be useful.

**Fig 4:**
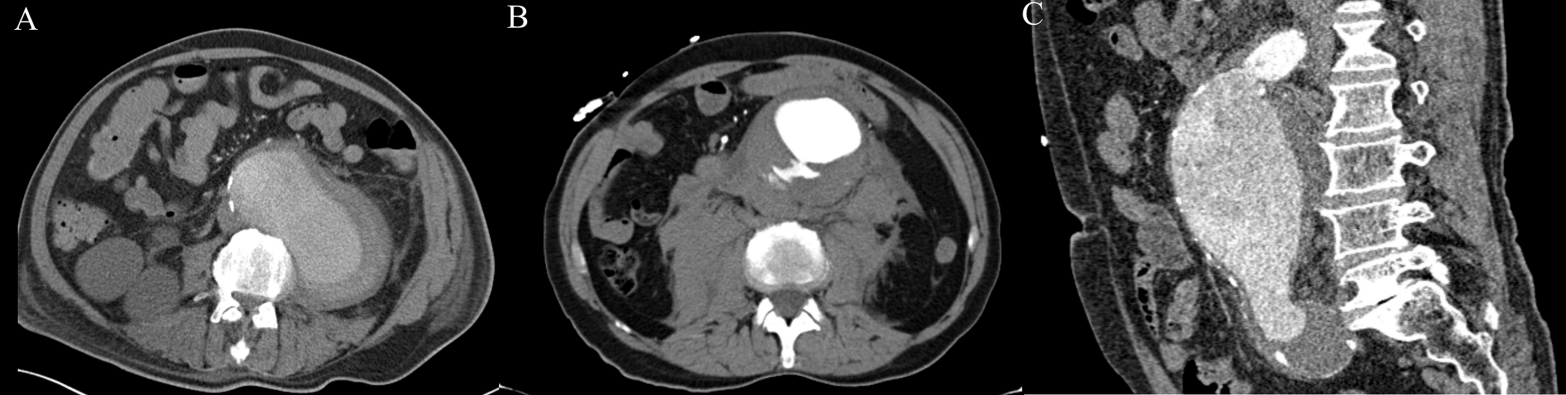
Examples of rAAA cases. A. AAA and hemorrhagic region could not be distinguished, this case was excluded. B. Contrast extravasation into the ILT, with manual correction a sufficient estimation of pre-rupture state could be acquired. C. Tortuous proximal aneurysm inlet with a decrease in contrast in the aneurysmal lumen and an iliac artery aneurysm, with much manual correction an segmentation could be made.

This study has some limitations. In all subjects endovascular infrarenal aneurysm repair was done. Therefore the size of the aAAA was skewed towards the threshold to treat (5.5cm) and thus these AAA already posed a higher risk of rupture. Additionally, the geometries included in this study are geometries suitable for endovascular repair without branches; i.e. an infrarenal AAA with a proper landing zone, sufficient iliac access and limited tortuosity. A quarter of the ruptured geometries analyzed (examples in Fig 4), and two percent of the asymptomatic geometries could not be accurately extracted. Consequently, FEA was not possible. As PWS increases at higher curvatures these cases indicate a possible benefit for the biomechanical analysis over the maximum diameter. However, the segmentation methods should first be optimized to be able to include all cases.

Previous studies used both pre-rupture and post-rupture geometries[9,15,25,28,33–35]. In these studies it was assumed that biomechanics of post-ruptured could still represent the pre-rupture situation. However, it can be argued that the rupture causes a significant geometrical change in the aneurysm. As a consequence the extracted post-rupture geometry might not result in a realistic estimation of the pre-rupture biomechanical indices. Nonetheless, no study could be found confirming or disapproving this statement. The comparison between aAAA and sAAA is therefore more promising for assessing the clinical usability of biomechanical analysis. However, larger cohorts are needed to confirm the results of this study.

The outcome was not blinded from the observer during the analysis. This is could have resulted in a bias, especially in the aAAA versus sAAA comparison. However, apart from the segmentation the complete analysis process was automated. Therefore bias could only occur when manual correction of the segmentation was needed. During this step both groups were treated as equally as possible. Apart of the segmentation the analysis process was automated and thus bias can only occur when manual correction of the segmentation is needed. As the software showed similar maximum diameters between the experienced radiologist and software for both aAAA and sAAA, the bias is likely to be minimal.

During this study the biomechanical showed similar AUC values for predicting rupture compared to the maximum diameter. The previous study of Fillinger et al. showed similar AUC-values, but the PWS AUC-value was significantly higher (0.88 versus 0.74) compared to the maximum diameter.[25]. Contrary to Fillinger et al. this study included the ILT and a patient specific blood pressure, both influence the wall stress.[36–44] Therefore, it is anticipated that the used model better represents the local geometry and physiology. However, this model did not include calcifications, which could also impact wall stress, especially at the border of the calcified plaque.[36,45,46] Notwithstanding, determining the exact position of the calcified plaque is difficult due to partial volume effect and luminal contrast enhancement. Thus including calcifications into the model will require an additional non-contrast CT or a validated method to quantify and locate the calcified volume.

Furthermore, other assumptions were made, such as a homogenous ILT composition and a uniform wall thickness. These factors might differ between aAAA and rAAA and subsequently might result in a similar PWS and PWRI in the diameter matched cases.

To conclude, this study showed no added value for biomechanical indices in AAA risk assessment. Clinical applicability is reduced by the complexity of the analysis However, as symptomatic aneurysms show a trend towards higher biomechanical indices with similar diameters the indices may provide additional information about aneurysm growth and development, but larger (prospective) studies are needed to truly evaluate the clinical usability.
